# Immunotherapy with 4-1BBL-Expressing iPS Cell‐Derived Myeloid Lines Amplifies Antigen-Specific T Cell Infiltration in Advanced Melanoma

**DOI:** 10.3390/ijms22041958

**Published:** 2021-02-16

**Authors:** Haruka Kuriyama, Satoshi Fukushima, Toshihiro Kimura, Hisashi Kanemaru, Azusa Miyashita, Etsuko Okada, Yosuke Kubo, Satoshi Nakahara, Aki Tokuzumi, Yuki Nishimura, Ikko Kajihara, Katsunari Makino, Jun Aoi, Shinichi Masuguchi, Hirotake Tsukamoto, Takashi Inozume, Rong Zhang, Tetsuya Nakatsura, Yasushi Uemura, Satoru Senju, Hironobu Ihn

**Affiliations:** 1 Department of Dermatology and Plastic Surgery, Faculty of Life Sciences, Kumamoto University, Kumamoto 860-8556, Japan; Haruka.kuriyama.toto@gmail.com (H.K.); chappie0_157@yahoo.co.jp (T.K.); hisashikanemaru@gmail.com (H.K.); miyasi-0530@live.jp (A.M.); etsuko2425069@gmail.com (E.O.); yousuke2056r@yahoo.co.jp (Y.K.); eternal_22year_old_md_alternate@yahoo.co.jp (S.N.); akin1022_b@yahoo.co.jp (A.T.); jjnnrzry122@gmail.com (Y.N.); kajiderma@gmail.com (I.K.); katsuderma@gmail.com (K.M.); junjunaoi@gmail.com (J.A.); masu_p999@yahoo.co.jp (S.M.); ihn-der@kumamoto-u.ac.jp (H.I.); 2 Department of Immunology, Graduate School of Medical Sciences, Kumamoto University, Kumamoto 860-8556, Japan; htsukamo@kumamoto-u.ac.jp; 3 Department of Dermatology, Graduate School of Medicine, Chiba University, Chiba 260-8677, Japan; inozumet@gmail.com; 4 Division of Cancer Immunotherapy, Exploratory Oncology Research & Clinical Trial Center, National Cancer Center (NCC), Kashiwa 277-8577, Japan; rzhang@east.ncc.go.jp (R.Z.); tnakatsu@east.ncc.go.jp (T.N.); yuemura@east.ncc.go.jp (Y.U.); 5 Department of Immunogenetics, Graduate School of Medical Sciences, Kumamoto University, Kumamoto 860-8556, Japan; senjusat@gpo.kumamoto-u.ac.jp

**Keywords:** iPS cells, 4-1BBL, CXCR6, melanoma, immune cell therapy

## Abstract

We have established an immune cell therapy with immortalized induced pluripotent stem-cell–derived myeloid lines (iPS-ML). The benefits of using iPS-ML are the infinite proliferative capacity and ease of genetic modification. In this study, we introduced 4-1BBL gene to iPS-ML (iPS-ML-41BBL). The analysis of the cell-surface molecules showed that the expression of CD86 was upregulated in iPS-ML-41BBL more than that in control iPS-ML. Cytokine array analysis was performed using supernatants of the spleen cells that were cocultured with iPS-ML or iPS-ML-41BBL. Multiple cytokines that are beneficial to cancer immunotherapy were upregulated. Peritoneal injections of iPS-ML-41BBL inhibited tumor growth of peritoneally disseminated mouse melanoma and prolonged survival of mice compared to that of iPS-ML. Furthermore, the numbers of antigen-specific CD8^+^ T cells were significantly increased in the spleen and tumor tissues treated with epitope peptide-pulsed iPS-ML-41BBL compared to those treated with control iPS-ML. The number of CXCR6-positive T cells were increased in the tumor tissues after treatment with iPS-ML-41BBL compared to that with control iPS-ML. These results suggest that iPS-ML-41BBL could activate antigen-specific T cells and promote their infiltration into the tumor tissues. Thus, iPS-ML-41BBL may be a candidate for future immune cell therapy aiming to change immunological “cold tumor” to “hot tumor”.

## 1. Introduction

In recent years, treatment for advanced melanoma has drastically changed, and prolonged survival durations are expected with cancer immunotherapy [1,2,3]. Cancer immunotherapy attempts to activate an antitumor immune response by altering signaling of the immunostimulatory or immunosuppressive molecules. Immune checkpoint inhibitors, such as anti-PD-1 antibodies and anti-CTLA-4 antibodies, show clinical effects in about 30–40% of the patients with advanced melanoma. However, some patients do not respond to these immune checkpoint inhibitors. One of the reasons is believed that tumor-infiltrating lymphocytes (TILs) cannot be seen in the tumor microenvironment of a certain number of patients [4]. Thus, another approach that can promote antigen-specific T cell infiltration is needed in these patients. Adoptive cell transfer (ACT) shows the most significant anticancer activity against malignant melanoma compared to other immune cell therapies [5]. However, such customized therapies take time and cost to prepare cells, and require advanced techniques. To overcome these problems, we have established an immune cell therapy with induced pluripotent stem (iPS) cell-derived myeloid lines (iPS-ML). The benefits of using iPS-ML are its infinite proliferative capacity and ease of genetic modification. We have previously used iPS-ML as effector cells and reported the effectiveness of type I interferon-expressing iPS-ML against some cancers [6,7]. Type I interferon delivery by iPS-ML elicits antitumor immunity via XCR1^+^ dendritic cells [8]. Furthermore, iPS-ML can act as dendritic cell-like antigen-presenting cells [9]. In this study, we introduced 4-1BBL gene to iPS-ML to enhance the antigen presentation ability of iPS-ML. As an inducible receptor of the TNF superfamily, 4-1BB is expressed on activated T lymphocytes, including cytotoxic T lymphocytes (CTL) [10], and its ligand—4-1BBL—is expressed on the surface of the antigen presenting cells (APC) [10,11]. The interaction between 4-1BB and 4-1BBL provides co-stimulatory signals for T-cell activation [12,13,14]. In initial studies, agonistic 4-1BB antibodies showed antitumor effects by activating both CD4^+^ and CD8^+^ T cells in sarcoma mouse models and increased CD8^+^ TILs in pancreatic cancer [15,16]. Agonistic antibodies against 4-1BB were subsequently found to be effective in reducing multiple tumors in mouse models of melanoma, glioblastoma, and lymphoma [17,18,19]. Today, agonistic 4-1BB antibodies have entered clinical trials. The agonistic anti-human 4-1BB IgG4 antibody urelumab (BMS-663513) caused dose-dependent hepatitis in patients [20,21]. Further, the anti-human 4-1BB IgG2 utomilumab (PF-05082566) was found to be safer, but less effective. Therefore, we considered the application of 4-1BB, in a method other than antibody preparation, and introduced 4-1BBL (CD137L) gene into iPS-ML. In this study, we investigated whether 4-1BBL introduced into iPS-ML could activate antigen-specific T cells and promote T cell infiltration into tumor tissues in a mouse melanoma model.

## 2. Results

### 2.1. Characterization of Mouse iPS-ML and iPS-ML-41BBL

**Figure 1:**
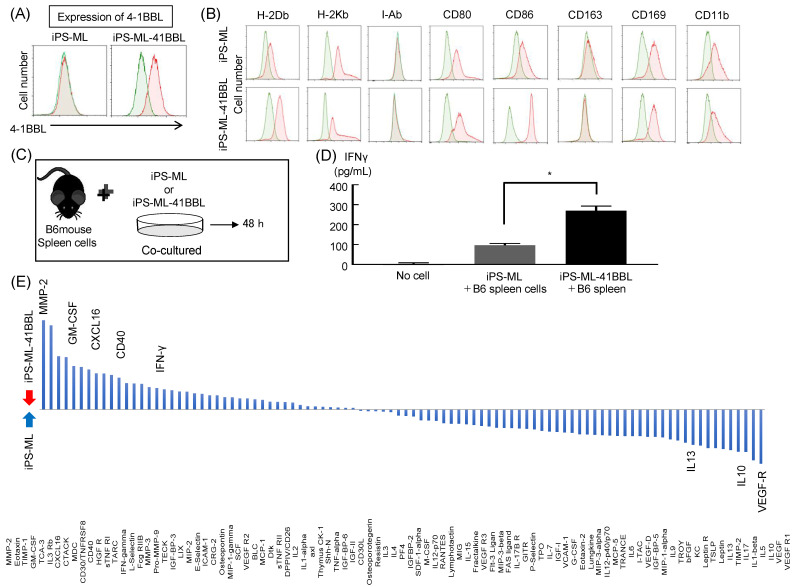
Generation of 4-1BBL-expressing iPS-ML (iPS-ML-41BBL). (**A**) Flow cytometry analysis of the expression of the cell surface molecule, 4-1BBL. The staining profiles of an isotype-matched control mAb (green lines) and the 4-1BBL mAb (red lines). (**B**) Flow cytometry for the expression of the cell surface molecules—HLA class I (H-2Db, H-2Kb), class II (I-Ab), co-stimulatory molecules (CD80, CD86,), and macrophage and dendritic cell markers (CD163, CD169, CD11b) on iPS-ML and iPS-ML-41BBL. The staining profiles of an isotype-matched control mAb (green lines) and the 4-1BBL mAb (red lines). (**C**) The model of sampling schedule for IFNγ ELISA and cytokine array. (**D**) Effects of iPS-ML expressing IFNγ on no cells, iPS- ML, and iPS-ML-41BBL. Expression of IFNγ was analyzed using ELISA. (**E**) Relative expression levels (iPS-ML-41BBL/iPS-ML) of 96 cytokines in the supernatants from cocultured B6 mouse spleen cells and iPS-ML or iPS-ML-41BBL. * *p* < 0.05.

First, we genetically modified and established 4-1BBL-overexpressing iPS-ML (iPS-ML-41BBL). After transduction of the 4-1BBL gene and selection by cell sorter, iPS-ML were observed with high 4-1BBL expression on their surface by flow cytometry (Figure 1A). We examined the expression of cell surface molecules between iPS-ML and iPS-ML-41BBL. The expression of the co-stimulatory molecule CD86 was upregulated in iPS-ML-41BBL compared to that in iPS-ML (Figure 1B).

### 2.2. Cytokine Analysis after Coculture of iPS-ML and Mouse Spleen Cells

We analyzed cytokines produced by the mouse spleen cells that were cocultured with iPS-ML or iPS-ML-41BBL (Figure 1C). First, we evaluated the expression of IFN-γ using specific ELISA, as IFN-γ is an essential cytokine for T cell immunity [22]. IFN-γ was significantly increased in the iPS-ML-41BBL group at 48 h after coculturing (Figure 1D). Next, we analyzed the expression of 96 cytokines under the same conditions. Immune-active cytokines, such as IFN- γ, IL-2, matrix metalloproteinase-2 (MMP-2), and CXCL16 were upregulated in iPS-ML-41BBL compared to in iPS-ML (Figure 1E). IL-2 is an essential cytokine for T-cell survival. MMP-2 plays an important role in cell infiltration [23]. CXCL16 attracts Th1 and NK T cells [24]. Furthermore, immunosuppressive cytokines, such as IL10, IL6, IL13, and VEGF were downregulated in iPS-ML-41BBL compared to in iPS-ML (Figure 1E).

### 2.3. iPS-ML-41BBL Inhibits Peritoneal Dissemination of Mouse Melanoma

**Figure 2:**
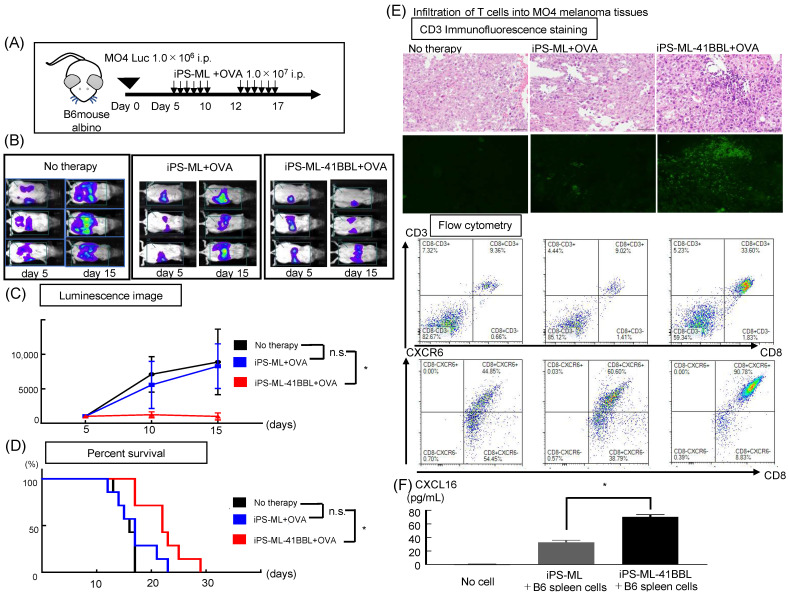
Effects of iPS-ML-41BBL against MO4 melanoma in vivo. (**A**) The model of treatment schedule; luciferase-expressing MO4 cells are intraperitoneally injected into B6-albino mice (1.0 × 10^6^ cells/mouse). After 5 days, the mice were randomly divided into control (*n* = 8), OVA peptide-pulsed iPS-ML (*n* = 8), and OVA peptide-pulsed iPS-ML-41BBL (*n* = 8) treatment groups. The mice in the OVA peptide-pulsed iPS-ML or OVA peptide-pulsed iPS-ML-41BBL groups were injected with 1 × 10^7^ cells/mouse/injection on days 5–10 and 12–17. (**B**) Luminescence images showing tumor growth. (**C**) Plots showing fold change in tumor-associated luminescence from day 5. (**D**) The Kaplan-Meier plot of the overall survival and median survival time (MST). * *p* < 0.05. (**E**) Infiltration of T cells into MO4 melanoma tissues. The treatment and sampling schedule are the same as shown in Figure 2A. The immunofluorescence staining was analyzed using a fluorescence microscope BZ-X700. T cell marker CD3 was stained by Alexa Fluor 488. FCM analysis of dissociated cells obtained from tumor tissues harvested on day 18. (**F**) The sampling schedule is the same as that shown in Figure 1C. Effects of iPS-ML expressing CXCL16 on no cells, iPS-ML, and iPS-ML-41BBL. Expression levels of CXCL16 were analyzed using ELISA. n.s. = not significant, * *p* < 0.05.

We examined the anticancer effects of iPS-ML-41BBL against disseminated mouse melanoma by in vivo imaging. We hypothesized that iPS-ML could present antigens to T cells and promote the antigen-specific cytotoxic activities of T cells. We evaluated antigen-specific tumor immunity induced by iPS-ML-41BBL using the model antigen, ovalbumin (OVA) (Figure 2A). An anticancer effect against OVA-expressing mouse melanoma (MO4) was not observed in the OVA peptide-pulsed iPS-ML group (*n* = 8) at day 15, and the survival time was slightly prolonged compared to that in untreated group (*n* = 8). However, in the OVA peptide-pulsed iPS-ML-41BBL–treated mice group (*n* = 8), extremely strong anticancer effects were observed (Figure 2B,C). Furthermore, the OVA peptide-pulsed iPS-ML-41BBL–treated group showed significantly prolonged survival compared to that in other groups in the mice (Figure 2D).

### 2.4. iPS-ML-41BBL Induce Changes in TILs

In tumor-bearing mice—after 5 days of OVA peptide-pulsed iPS-ML therapy—we examined the infiltration of T cells into MO4 melanoma tissues using immunofluorescence staining and flow cytometry (FCM). Immunofluorescence staining showed that a significantly higher number of TILs infiltrated in the tumor tissues treated with iPS-ML-41BBL than in untreated tumor tissues or those treated with iPS-ML (Figure 2E). We examined the distribution of T cells using FCM. The proportion of CD8^+^ T cells in tumor tissues was increased in the group treated with OVA peptide-pulsed iPS-ML-41BBL compared to those treated with iPS-ML (Figure 2E). Furthermore, increased infiltration of CD8^+^ T cells expressing the chemokine receptor CXCR6—which is the receptor for CXCL16—was observed in the tumor tissues treated with iPS-ML-41BBL compared to those treated with iPS-ML (Figure 2F). These results suggest that iPS-ML-41BBL has the ability to induce infiltration of CTLs into the tumor.

### 2.5. In Vivo Priming of iPS-ML-41BBL Therapy

**Figure 3:**
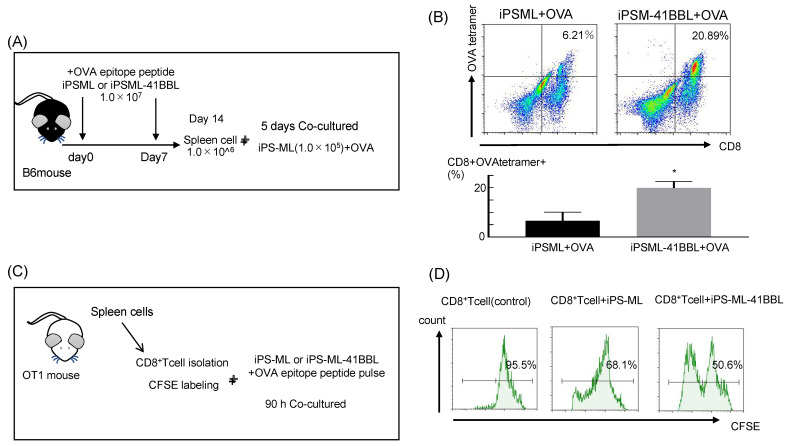
Antigen-presentation capacity of iPS-ML-41BBL (**A**) Model of treatment and sampling schedule; non-tumor–bearing B6 mice were intraperitoneally injected with OVA peptide-pulsed iPS-ML or OVA peptide-pulsed iPS-ML-41BBL on days 0 and 7, and the spleen tissues were harvested on day 14. Cells (1 × 10^6^ cells/well) were cocultured with OVA peptide-pulsed iPS-ML (1 × 10^5^ cells/well). After culturing for 5 days, the floating cells were collected and analyzed by FCM. (**B**) FCM analysis of OVA-specific CD8^+^ T cells. * *p* < 0.05. (**C**) Carboxyfluorescein succinimidyl ester (CFSE)-labeled CD8^+^ T cells from OT-I transgenic mice and no cell, OVA peptide-pulsed iPS-ML or OVA peptide-pulsed iPS-ML-41BBL were cocultured at 1:1 dilutions. (**D**) Proliferation of OT-I cells was assessed after 72 h by flow cytometry. * *p* < 0.05.

Next, we examined the ability of iPS-ML-41BBL to activate antigen-specific T cells using OVA tetramers. The mice were immunized in OVA-pulsed iPS-ML or iPS-ML-41BBL (Figure 3A). After 14 days of immunization with OVA peptide-pulsed iPS-ML or iPS-ML-41BBL, the spleen cells were harvested and cocultured with OVA peptide-pulsed iPS-ML for 5 days. The OVA-specific CD8^+^ T cells were significantly increased in mice treated with OVA peptide-pulsed iPS-ML-41BBL compared to those treated with OVA peptide-pulsed iPS-ML. The percentage of CD8^+^ T cells increased from 6.21 to 20.89% (Figure 3B). These results indicate that iPS-ML-41BBL efficiently promotes activation of antigen-specific CTLs compared to iPS-ML, in vivo.

### 2.6. Antigen Presenting Ability of the iPS-ML-41BBL

Next, we analyzed the antigen-presentation capacity of iPS-ML-41BBL using OT-I transgenic mice (Figure 3C). T cells harvested from OT-1 transgenic mice only recognize the complex of OVA epitope peptide and MHC [25]. Significantly higher proliferation levels were observed in the OT-I CD8^+^ cells cocultured with OVA peptide-pulsed iPS-ML-41BBL than in those cocultured with OVA peptide-pulsed iPS-ML (Figure 3D).

### 2.7. Infiltration of iPS-ML in Established Tumor Tissue 

**Figure 4:**
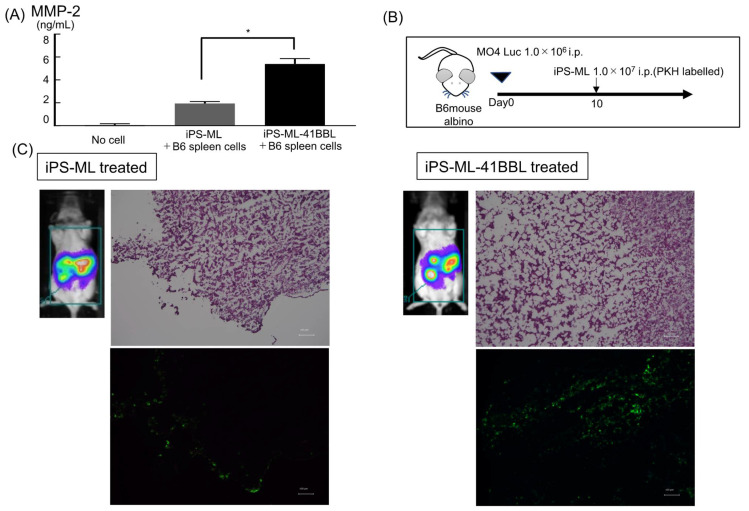
Infiltration of iPS-ML-41BBL in established tumor tissues in the mouse peritoneal cavity. (**A**) Analysis of levels of MMP-2 in the supernatant obtained from the cocultured spleen cells and iPS-ML or iPS-ML-41BBL using MMP-2–specific ELISA. The sampling schedule is the same as in Figure 1C. (**B**) The model of treatment schedule; luciferase-expressing MO4 cells were intraperitoneally injected into B6-albino mice (1.0 × 10^6^ cells/mouse). (**C**) Luminescence imaging analysis in mice. Scale bar: 100 µm. Tumor tissues were analyzed using a fluorescence microscope BZ-X700. * *p* < 0.05.

As mentioned above, the cytokine array revealed upregulation of MMP-2 in the supernatant obtained from cocultured iPS-ML-41BBL and spleen cells (Figure 1C). We also confirmed this result using MMP-2 specific ELISA (Figure 4A). MMP-2 improves the infiltration abilities of both tumor cells and macrophages [26,27]. Thus, we evaluated the intraperitoneally administered iPS-ML or iPS-ML-41BBL infiltrated tumor tissue that had been established in the peritoneal cavity of mice. Luciferase-expressing MO4 (MO4-Luc) (1 × 10^6^ cells) were intraperitoneally injected into B6-albino mice. After 10 days, luminescence imaging analysis was performed (Figure 4B,C). Mice engrafted with melanoma cells were intraperitoneally injected with iPS-ML labeled with a green fluorescent dye, PKH67. The mice were sacrificed the following day and subsequently dissected to determine the location of the injected tumor cells and iPS-ML using fluorescence analysis. Significantly higher infiltration of iPS-ML-41BBL into melanoma tissues was observed than that of iPS-ML.

### 2.8. ES-ML (129)-41BBL Suppresses the Peritoneal Dissemination of MO4 Melanoma

**Figure 5:**
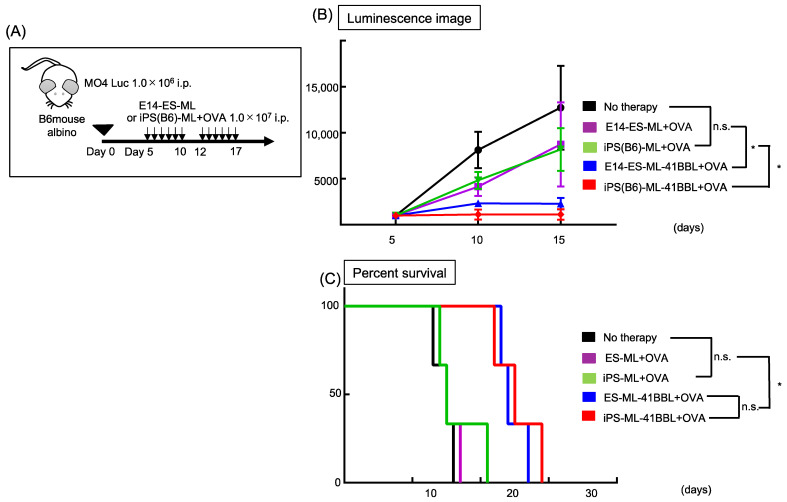
Effects of ES-ML-41BBL against MO4 melanoma in vivo. (**A**) The model of treatment schedule. (**B**) Luminescence images showing tumor growth. (**C**) Plots showing fold change in tumor-associated luminescence at day 5. Kaplan-Meier plot of overall survival and median survival time (MST). n.s. = not significant * *p* < 0.05.

In future clinical applications, we plan to use human allogenic iPS-ML to treat patients with advanced melanoma. Therefore, we tested whether allogenic stem cell-derived myeloid lines could inhibit tumor growth in the mouse melanoma model (Figure 5A). We examined the anticancer effects of embryonic stem (ES) cell-derived myeloid lines (ES-ML) established from the 129Sv mouse strain, which has a different MHC class II compared to C57BL6 mouse. MO4 is a syngeneic tumor of C57BL6 mouse; therefore, these experiments mimic allogenic situations. The anticancer effects of ES-ML (129)-41BBL against MO4 were inferior to those of iPS-ML (B6)-41BBL group (*n* = 3) at day 15 (Figure 5B). However, OVA peptide-pulsed ES-ML (129)-41BBL showed stronger anticancer effects than the no-therapy group (*n* = 3). Furthermore, there was no predominant difference between iPS-ML and ES-ML in the survival curve, which was improved compared to the survival in other groups (Figure 5C). These results suggest that allogenic ES-ML could show anticancer effects and that 4-1BBL-targeted therapeutic strategies also work in allogenic environments.

### 2.9. Autoimmune Reactions after iPS-ML-41BBL Therapy

To confirm whether iPS-ML therapy induces autoimmune reactions, we performed immunohistochemical analysis to investigate the infiltration of CD3^+^ T cells into the kidney, liver, and lung tissues of mice treated with iPS-ML or iPS-ML-41BBL (Figure S1). No significant differences in the number of infiltrating CD3^+^ T cells and morphological abnormalities were observed between the treated and untreated mice (Figure S1). These results suggest that iPS-ML-41BBL therapy may not evoke any immune-related adverse events at the doses administered in this study.

## 3. Discussion

This study confirmed the efficacy of 4-1BBL–expressing iPS-ML in a mouse melanoma model. Peritoneal injections of iPS-ML-41BBL significantly inhibited the tumor growth of peritoneally disseminated melanoma and prolonged survival compared to that of iPS-ML. Furthermore, the number of antigen-specific CD8^+^ T cells was significantly increased in the tumor tissues after treatment with epitope peptide-pulsed iPS-ML-41BBL compared to that with iPS-ML. These results suggest that iPS-ML-41BBL could activate antigen-specific T cells and promote their infiltration into the tumor tissues and inhibit tumor growth.

Teng et al. reviewed types of tumor microenvironments to tailor cancer immunotherapeutic modules [4]. Based on the presence of TILs and PD-L1 expression, they categorized tumor microenvironments into four categories as follows: type I (adaptive immune resistance), type II (immunologic ignorance), type III (intrinsic induction), and type IV (tolerance). This proposed framework of stratifying tumors is simplistic but provides a platform to discuss immunotherapeutic strategies targeting the four different tumor microenvironments. Among advanced melanomas, approximately 38% of the patients present with the type I tumor microenvironment, and are responders to checkpoint blockades [28,29]. Thus, approximately 60% of the patients need other therapeutic approaches to change an immunologically “cold tumor” to “hot tumor.” In type II and III tumors, TILs are missing; therefore, an approach to attract T cells into tumors is necessary. As shown in this study, iPS-ML-41BBL could activate antigen-specific T cells and promote their infiltration into the tumor tissues. In type IV tumors, TILs exist; however, immune regulatory cells, such as M2 macrophages, regulatory T cells, and myeloid derived suppressor cells are dominant. Therefore, an approach to change the cell phenotype from regulatory to inflammatory is needed. We previously showed that type I IFN introduced iPS-ML increased the expression of CD169, a marker of M1 macrophages, that can activate antitumor immunity. We also confirmed the infiltration of iPS-ML expressing type I IFN into the tumor nests [7]. Taken together, we believe that immune cell therapy with both iPS-ML-41BBL and iPS-ML expressing type I IFN can overcome the immune-resistant tumor microenvironments, such as types II, III, and IV.

There are a few technical problems to be addressed before initiating clinical trials with iPS-ML. One is the risk of using immortalized cells for the treatment of melanoma. iPS-ML were generated from iPS cells by introducing c-*MYC* to obtain the proliferative capacity for the purpose of automatic mass culture and serum-free growth. This will enable cost reduction and strict quality control when iPS-ML are largely used in future clinical applications. However, the risk of using immortalized cells such as development of leukemia and other malignant tumors must be minimized. Therefore, we are in the process of preparing iPS-ML–introduced “safety switch” using some suicide genes, such as HSV-TK [30]. Furthermore, we are planning to use allogenic iPS cells from the iPS bank in future clinical applications. In this study, we showed that allogenic ES-ML could confer anticancer effects, and that the 4-1BBL–targeted therapeutic strategy also worked in an allogenic environment. The immune system excludes allogenic cells completely by recognizing their unmatched HLA. Even if the iPS-ML with all HLA class I and class II matched are used, allogenic iPS-ML will be excluded by recognizing minor antigens by the immune system. In this strategy, iPS-ML is expected to be excluded after activating T cells and changing the tumor microenvironment.

In conclusion, we propose that iPS-ML-41BBL may be a candidate for future immune cell therapy aiming to change the immunologically “cold tumor” to “hot tumor”.

## 4. Materials and Methods

### 4.1. Cells and Culture Conditions

The mouse melanoma cell line B16-BL6 was purchased from the ATCC (Manassas, VA, USA) and ovalbumin (OVA)-expressing melanoma MO4 cells were kindly provided by Dr. Kenneth L. Rock, Department of Pathology, UMass Medical School (Worcester, MA, USA). These cells were cultured in Dulbecco’s Modified Eagle Medium (DMEM; high glucose; Wako) supplemented with 20% FBS and 1% antibiotic-antimycotic 100 U/mL penicillin (Life Technologies, Carlsbad, CA, USA). The cells were transduced with a lentivirus vector encoding the firefly luciferase gene, as described previously for the analysis based on luciferase activity [9]. ES-ML cells were generated from E14 ES cells derived from the 129Sv mouse and maintained as described previously [31]. C57BL/6 genetic background iPS cells (2A-4F-100) were generated as described previously [32]. iPS-ML were generated and maintained as described previously [33]. Briefly, E14 ES cells and B6 iPS cells were seeded onto a feeder cell layer of OP9 mouse bone marrow stromal cells. On day 5 or 6, cells were harvested, reseeded onto fresh OP9 cell layers, and cultured in the presence of granulocyte-macrophage colony-stimulating factor (1000 U/mL). On days 13 and 14, myeloid lineage cells were recovered and transduced with a lentiviral expression vector CSIIEF containing c-*MYC* to establish the ES-ML or iPS-ML. The H-2K^b^ restricted mouse OVA_257–264_ peptide (MBL; SIINFEKL) and H-2K^b^ restricted SIY peptide (MBL; SIYRYYGL) were used for in vivo treatment.

### 4.2. Mice

Female C57BL/6J and C57BL/6 albino B6(Cg)-Tyr^c-2J^/J (B6-albino; JAX stock #000058) mice (6 weeks old) (Jackson Labs, Bar Harbor, ME, USA) were used and housed in a pathogen-free environment. OT-1 transgenic mice were obtained from the Jackson Labs [34].

### 4.3. Flow Cytometry (FCM)

Cell samples were treated with a mouse FcR-blocking reagent (Miltenyi Biotec, Bergisch Gladbach, Germany) for 10 min, stained with the fluorochrome-conjugated monoclonal antibodies (mAbs) for 20 min, washed twice with PBS, and analyzed using a Novocyte flow cytometer (ACEA Bioscience, San Diego, CA, USA). The following mAbs conjugated with FITC, PE, APC, or PerCP Cy3.3 and the isotype controls were purchased from BioLegend (San Diego, CA, USA), Thermo Fisher Scientific (Carlsbad, CA, USA), R&D systems (Minneapolis, MN, USA) and Miltenyi Biotec: anti-mouse 4-1BBL (TKS-1, rat IgG2a), anti-mouse H-2K^b^ (AF6-88.5, mouse IgG2a), anti-mouse H-2D^b^ (KH95, mouse IgG2b), anti-mouse I-A^b^ (REA528, recombinant human IgG1), anti-mouse CD80 (16-10A1, Armenian Hamster IgG), anti-mouse CD86 (GL1, rat IgG2a), anti-mouse CD163 (TNKUPJ, rat IgG2a), anti-mouse CD169 (3D6.112, rat IgG2a), anti-mouse CD11b (M1/70, rat IgG2b), anti-mouse CD3 (17A2, Rat IgG2b), anti-mouse CD4 (RM4-5, Rat IgG2a or GK1.5, rat IgG2b), anti-mouse CD8a (53-6.7, rat IgG2a), and anti-mouse CXCR6 (#221002, ratIgG2b).

### 4.4. Generation of Genetically Modified 4-1BBL-Expressing iPS-ML (iPS-ML-41BBL)

cDNA fragments encoding the complete mouse 4-1BBL gene were obtained by reverse transcription PCR (RT-PCR) from the mouse spleen cells. The 4-1BBL gene was transferred to a mammalian expression vector and transduced with a lentivirus vector (pLVX-IRES-mCherry; TaKaRa, Shiga, Japan). To select cells stably expressing the transgenes, the mCherry-positive cells were collected using the SH800 cell sorter (SONY) and cultured in the same medium as that used for culturing iPS-ML. Expression of 4-1BBL was quantified and the differences between the expression of other cell-surface molecules in iPS-ML and iPS-ML-41BBL cells were analyzed by FCM (Figure 1A).

### 4.5. Cytokine Production, ELISA, and Cytokine Array of Cocultured iPS-ML and the Mouse Spleen Cells

We analyzed the expression of type I IFN, CXCL16, and MMP-2 using the following ELISA kits: type I IFN (MIF00, R&D systems), CXCL16 (ab100677, Abcam, Cambridge, UK), and MMP-2 (MMP200, R&D systems). We analyzed the expression of 144 cytokines in the supernatants of cocultured B6 mouse spleen cells and iPS-ML or iPS-ML-41BBL. The B6 mouse spleen tissues were harvested and dissociated into single-cell suspensions using the gentleMACS Dissociator (Miltenyi Biotec). Red blood cells (RBCs) were lysed using RBC lysis buffer (Roche). The spleen cells (1 × 10^6^ cells/well) and iPS-ML or iPS-ML-41BBL (1 × 10^5^ cells/well) were cocultured for 5 days in 6-well culture plates in RPMI-1640 medium supplemented with 10% FBS, 100 U/mL recombinant mouse IL-2 (R&D Systems), and 50 μM 2-mercaptoethanol. The supernatants were then sampled and assayed using the mouse cytokine array C-series 2000 kit (Ray Biotech, Norcross, GA USA).

### 4.6. In Vivo Antitumor Activity of iPS-ML against MO4

B6-albino mice were intraperitoneally injected with luciferase-expressing MO4 cells (MO4-Luc) (1 × 10^6^ cells/mouse). On day 5, the mice were subjected to luminescence image analysis to identify tumor establishment. Mice with established tumors were randomly divided into control and treatment groups. The mice in the treatment group were intraperitoneally injected with OVA peptide-pulsed iPS-ML or OVA peptide-pulsed iPS-ML-41BBL (total 1 × 10^7^ cells/mouse for each injection) on day 6 of each week and continued for 2 cycles. iPS-ML or iPS-ML-41BBL were incubated for 2 h in medium containing 1 μg/mL of H-2K^b^ restricted mouse OVA_257-264_ peptide (MBL; SIINFEKL)(Gene tex, Irvine, CA, USA). All mice underwent bioluminescence analysis on days 5 and 10, to evaluate the effects of the treatment.

### 4.7. OVA Tetramer Assay

Non-tumor–bearing B6 mice were treated with peptide (OVA or control)-pulsed iPS-ML or iPS-ML-41BBL on days 0–4, and the spleen tissues were harvested on day 10. The SIY peptide (MBL; SIYRYYGL) was used as a control peptide. The isolated spleen cells were stained with PE-conjugated H-2K^b^ OVA tetramer (MBL) for 20 min, stained with CD3 and CD8a mAbs, and analyzed by FCM, as described above. Furthermore, 1 × 10^6^ spleen cells were re-stimulated in vitro by coculturing with OVA-pulsed iPS-ML (1 × 10^5^ cells/well) in 6-well culture plates. The culture medium used was same as that for the cytokine array. After culturing for 5 days, the floating cells were collected and analyzed by FCM.

### 4.8. Functional Analysis of TILs

MO4-Luc (1 × 10^6^ cells) were intraperitoneally injected into B6-albino mice on day 0. The mice were untreated or treated with 1 × 10^7^ cells of OVA peptide-pulsed iPS-ML or OVA peptide-pulsed iPS-ML-41BBL for 6 days per week and continued for 2 cycles. The tumor tissues were harvested after 2 cycles of treatment. Half of the tumor tissue was fixed in formalin, and the remaining tumor and spleen tissues were dissociated as described above. These tissues were used for subsequent analysis.

### 4.9. Immunofluorescence Staining

The formalin-fixed tumor tissues were embedded in paraffin, and 4-μm thick sections were obtained. The sections were stained with the primary antibodies—purified anti-mouse CD3 (CD3-12, rat IgG1) and anti-mouse Melan-A (EPR20380, rabbit IgG)—and the secondary antibodies—Alexa Fluor 488-conjugated anti-rabbit IgG and Alexa Fluor 594-conjugated anti-rat IgG. The antibodies were purchased from Abcam. The stained sections were treated with the TrueVIEW^TM^ autofluorescence quenching kit (VECTOR Laboratories, Burlingame, CA, USA), mounted with HardSet Antifade Mounting Medium with DAPI (VECTOR Laboratories), and analyzed using a BZ-X700 fluorescence microscope (KEYENCE, Osaka, Japan).

### 4.10. PKH67 Labeling

MO4-Luc (1.0 × 10^6^ cells) were intraperitoneally injected into B6-albino mice. After 10 days, luminescence imaging analysis was performed. Mice engrafted with melanoma cells were randomly divided into iPS-ML–treated or iPS-ML-41BBL–treated. The mice were intraperitoneally injected with iPS-ML or iPS-ML-41BBL cells (1 × 10^7^ cells/mouse) labeled with the green fluorescent dye PKH67 (Sigma, Kanagawa, Japan, PKH67 GL). Briefly, 1 × 10^7^ cells were suspended in 1.0 mL of Diluent C and stained by rapid admixing with a 20 μM working PKH67 solution, prepared by diluting 20 μL of 1 × 10^−3^ M ethanolic dye stock in 1.0 mL of Diluent C immediately prior to staining. The final staining concentration was 10 μM PKH67 for 5 × 10^6^ cells/mL. Staining was stopped after 3 min by the addition of 2 mL of FBS, and cells were washed 3 times with 5 mL of RPMI-1640 medium containing 10% FBS.

### 4.11. Antigen-Presentation Assays

Antigen-presentation assays were performed as previously described [35,36]. Ovalbumin-specific CD8^+^ OT-I cells were obtained from mouse spleens using a CD8^+^ T Cell Isolation Kit (Miltenyi Biotec). T cells were labeled with CFSE (BioLegend) according to the manufacturer’s instructions. For OT-I T-cell proliferation, CD8^+^ T cells from the splenocytes of OT-I transgenic mice were labeled with CFSE. iPS-ML or iPS-ML-41BBL were pulsed with OVA epitope peptide (1 μM for 2 h), except for cells in control wells. All the cells were then washed twice with PBS and cocultured with 5 × 10^4^ CFSE-labeled naïve OT-I transgenic CD8^+^ T cells at decreasing dilutions. The proliferation of OT-I cells was measured after 72 h by flow cytometry.

### 4.12. Statistical Analysis

Mann-Whitney *U* test was used to examine the differences between tumor-associated luciferase activities. Kaplan-Meier survival and Gehan-Breslow-Wilcoxon tests were used for comparing survival in tumor-bearing mice. Statistical analyses were performed using Prism software (GraphPad); *p* < 0.05 was considered to be statistically significant.

## Data Availability

Date is contained within the article or Supplementary Material.

## Supplementary Materials

The following is available online at https://www.mdpi.com/1422-0067/22/4/1958/s1, Figure S1: Schedule for the post-treatment autoimmunity assay. The treatment and sampling schedule are the same as in Figure 2A. The kidney, liver, and lung tissues were harvested on day 18. Results of immunohistochemical analyses after staining of the tumor tissues with anti-CD3 antibody are shown. Scale bars = 50 μm.
