# Supplementary material for: Immunotherapy with 4-1BBL-Expressing iPS Cell‐Derived Myeloid Lines Amplifies Antigen-Specific T Cell Infiltration in Advanced Melanoma
Source: Int J Mol Sci. 2021 Feb 16;22(4):1958. doi: 10.3390/ijms22041958 (PMC7920470; doi:10.3390/ijms22041958)
Supplement: Supplementary file 1 [file ijms-22-01958-s001.pdf]

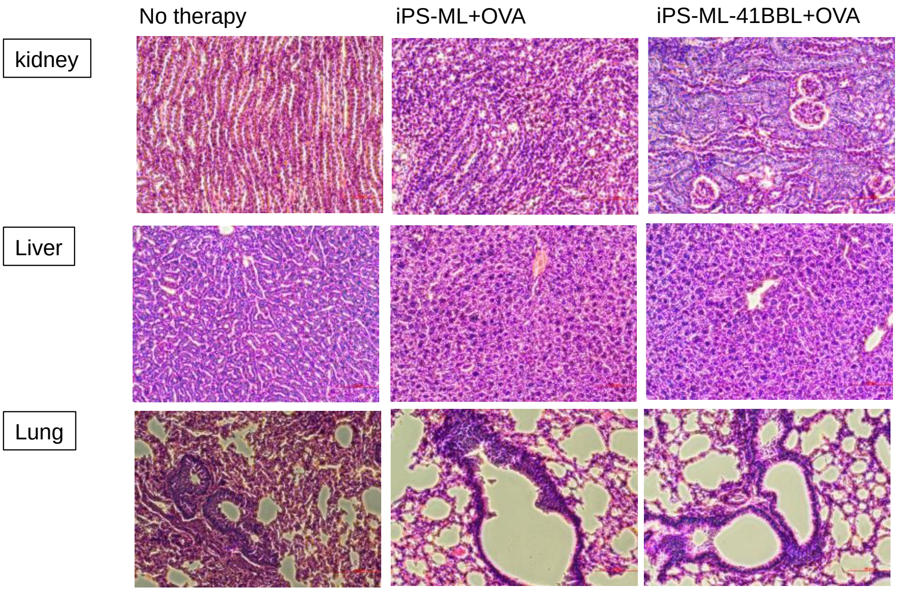


**Figure S1.** Schedule for the post-treatment autoimmunity assay. The treatment and sampling schedule are same as Figure 2A. The kidney, liver, and lung tissues are harvested on day 18. Results of immunohistochemical analyses after staining of the tumor tissues with anti-CD3 antibody are shown. Scale bars = 50 μm.
